# Indoor air pollution inequalities among children and adolescents in Germany: an analysis of repeated cross-sectional data from GerES and KiGGS

**DOI:** 10.1038/s41598-025-04278-9

**Published:** 2025-06-20

**Authors:** Jascha Wiehn, Sarah Tietjen, Florian Beese, Wolfram Birmili, Christiane Bunge, Anja Daniels, Annika Fernandez Lahore, Domenica Hahn, Marike Kolossa-Gehring, Ronny Kuhnert, Aline Murawski, Julia Waldhauer, Dario Zocholl, André Conrad

**Affiliations:** 1 Department of Environmental Hygiene, German Environment Agency (Umweltbundesamt), 14195 Berlin, Germany; 2 Department of Epidemiology and Health Monitoring, Robert Koch Institute, 13353 Berlin, Germany; 3 Department of Medical Biometrics, Informatics and Epidemiology, Faculty of Medicine, University of Bonn, 53127 Bonn, Germany

**Keywords:** Environmental social sciences, Risk factors

## Abstract

Indoor air pollution may harm child health. Indoor air pollution inequalities among children and adolescents is under-researched. We analyzed associations between equivalized disposable income, socioeconomic status, and history of migration with benzene, toluene, xylene, limonene, and formaldehyde among children and adolescents in Germany. Using pooled data from the German Environmental Survey (GerES IV, GerES V) and the German Health Interview and Examination Survey for Children and Adolescents (KiGGS Baseline, KiGGS Wave 2) (N = 1117, aged 3–14 years), six out of fifteen random intercept models revealed statistically significant findings. An increase of one standard deviation in equivalized disposable income was associated with 5% lower benzene concentrations (exp(ß): 0.95, 95% confidence interval [CI] 0.91, 0.99). Higher socioeconomic status was associated with a 10% decrease in benzene (exp(ß): 0.90, 95% CI 0.87, 0.94) and a 6% decrease in toluene (exp(ß): 0.94, 95% CI 0.89, 0.99). Having a parental history of migration was associated with 24% higher concentrations of formaldehyde (exp(ß): 1.24, 95% CI 1.07, 1.43) and 102% increased limonene concentrations (exp(ß): 2.02, 95% CI 1.61, 2.55). Subgroup analysis from urban municipalities showed only slight differences. Although results varied, they highlight that indoor air pollution is unequally distributed among children and adolescents in Germany.

## Introduction

Indoor air pollution refers to elevated concentration levels of pollutants in the indoor air with negative impacts on the human body^1^. Indoor air pollutants pose potential health risks for children and adolescents living in northern latitudes, given that they spend the largest part of their day indoors^2^. Compared to adults, young children are particularly susceptible to indoor air pollutants, because some of their organs, immune, and metabolic systems are still developing and they have higher breathing rates^3^. Benzene, toluene, xylene, and formaldehyde are typical volatile organic compounds (VOCs) with a high public health relevance, due to their toxicological properties and exposure-related potency^4^. The adverse health effects of exposure on children include impacts on the immune, neurological, and respiratory systems as well as an impact on the cognitive development^5^. Furthermore, epidemiological evidence indicates that prolonged exposure to benzene and formaldehyde may result in adverse health outcomes, including the development of cancer^6^. Limonene is another VOC present in indoor air that is of lower toxicological relevance. However, it can cause allergic and respiratory symptoms and supports the formation of secondary pollutants^4,7^.

The sources of VOCs are manifold^8^. Combustion and vaporisation processes emit the five aforementioned pollutants from a range of indoor and outdoor sources^9^. Typical sources include combustion engines or tobacco smoke in the case of benzene, industrial plants or paints in the case of toluene and xylene, hygiene products or cleaning agents in the case of limonene and building products or adhesives in the case of formaldehyde^10^. Outdoor sources of VOCs can be attributed to traffic, emissions from contaminated soil, industrial activities, or residential activity like barbecuing. The magnitude of exposure through these sources further depends on the physical properties of the dwelling (e.g., air exchange rate or occupancy density), and the activity patterns of the occupants (e.g., frequency of smoking indoors)^8^.

As many of the structural and behavioral sources mentioned above correlate with socioeconomic and -demographic factors such as household income^11^, it stands to reason that VOCs in household indoor air may vary accordingly. There are various mediation pathways in which socio-economic and -demographic factors might affect VOC concentrations. For example, a lower educational attainment might increase the likelihood of smoking^12^, while household occupants smoking indoors would then increase concentration levels of VOCs^13^. Another mediation path could lie in the fact that disadvantaged groups, e.g. through a history of migration, tend to experience discriminatory mechanisms at the housing market more frequently^14^. This might lead to residing in housing situations with higher ambient air pollution^15,16^, and therefore to higher indoor air pollution through outdoor-indoor exchange^17^.

In contrast to outdoor settings, socially unequal levels of indoor air pollution have rarely been addressed. An earlier scoping review of studies from high-income countries concluded that the most disadvantaged populations are disproportionately exposed to indoor air pollutants^18^. However, the research findings are quite heterogeneous, depending on the pollutant in question. While a large number of primary studies consistently demonstrated that higher household income is associated with lower levels of indoor particulate matter (PM)^19–23^, the effect directions were inconsistent when it came to the relation between income and VOCs^23,24^. Although some environmental justice studies reported variations in indoor air pollution across socioeconomic and -demographic groups of children and adolescents in various countries, including the United Kingdom^19^, Denmark^25^, Greece^26^, the United States^24^, South Korea^27,28^, and Spain^29^, a corresponding analysis specific to the German context has yet to be conducted.

Given the lack of evidence, our objective is to describe the distribution of the five VOCs mentioned above across a spectrum of socioeconomic and -demographic characteristics among children and adolescents in Germany. Specifically, we aim to answer the following primary research question: Are concentration levels of benzene, toluene, xylene, limonene, and formaldehyde associated with equivalized disposable income, socioeconomic status, or parental history of migration among children and adolescents aged between 3 and 14 years residing in Germany? We hypothesize that an increase in equivalized disposable income and socioeconomic status, or the absence of a parental history of migration, is associated with lower concentrations of benzene, toluene, xylene, limonene, and formaldehyde. We consider the derivation of descriptive measures of association to answer this research question as a descriptive task and not a causal one^30^.

As outdoor sources of air pollution, socially disadvantaged groups, or people with a history of migration tend to cluster in urban settings, it is possible that environmental inequalities are linked to the degree of urbanization^31^. Due to the critical role of urbanity in environmental justice research^32^, we furthermore explore whether the observed associations differ by municipality size. Such a comparison of indoor air pollution inequalities may reveal distinct disadvantages that can be addressed by future urban planning in Germany.

## Results

By merging the data from the German Environmental Survey for Children 2003–2006 (GerES IV), the German Environmental Survey for Children and Adolescents 2014–2017 (GerES V) and the respective corresponding datasets from the German Health Interview and Examination Survey for Children and Adolescents (KiGGS Baseline, KiGGS Wave 2), we obtained a pooled database with 4084 participants. After applying eligibility criteria for our subjects (see methods), the final study sample comprised 1117 children and adolescents aged 3 to 14 years.

### Descriptive analysis

Table 1 describes the characteristics of the pooled study sample and the GerES IV and GerES V samples. In the pooled data set, three quarters of the participants lived in municipalities with ≥ 20,000 inhabitants, the households had a mean equivalized disposable income of 1482.5 Euros (standard deviation [SD] = 847.1) and a mean socioeconomic status score of 12.5 (SD = 4.3). Supplementary Figure S1 shows that the equivalised disposable income is right-skewed, with a median of 1261 Euros. Eight percent of the participants had a parental history of migration, defined as both parents, or in case of single-parents the parent with whom the child lives, were born in another country than Germany. The geometric mean values of the air pollutant concentration in the households of the entire study population were between 1.6 µg/m^3^ (95% confidence interval [CI] 1.5, 1.7) for benzene and 23.2 µg/m^3^ (95% CI 21.6, 24.8) for formaldehyde.

**Table 1:** Sample characteristics of GerES IV, GerES V, and pooled data.

	GerES IV (N = 579)	GerES V (N = 538)	Pooled data (N = 1117)
Municipality size, N (%)			
< 20,000 inhabitants	195 (34.2)	84 (14.8)	279 (24.9)
≥ 20,000 inhabitants	384 (65.8)	454 (85.2)	838 (75.1)
Missings, N	0	0	0
Equivalized disposable income [Euro]			
AM (SD)	1161.2 (582.0)	1822.5 (945.7)	1482.5 (847.1)
Median	1061	1611	1261
Missings, N	6	1	7
Socioeconomic status			
AM (SD)	11.4 (4.2)	13.8 (4.1)	12.5 (4.3)
Missings, N	1	2	3
History of migration, N (%)			
No	539 (92.1)	487 (91.9)	1026 (92.0)
Yes	32 (7.9)	28 (8.1)	60 (8.0)
Missings, N	8	23	31
Benzene [µg/m^3^]			
GM (95% CI)	2.1 (1.8, 2.3)	1.2 (1.1, 1.4)	1.6 (1.5, 1.7)
Missings, N	34	43	77
Toluene [µg/m^3^]			
GM (95% CI)	14.8 (13.3, 16.6)	5.2 (4.5, 5.9)	8.9 (8.1, 9.9)
Missings, N	34	43	77
Xylene [µg/m^3^]			
GM (95% CI)	5.1 (4.6, 5.7)	3.4 (2.9, 3.9)	4.2 (3.8, 4.6)
Missings, N	34	43	77
Formaldehyde [µg/m^3^]			
GM (95% CI)	23.8 (22.5, 25.1)	22.5 (19.8, 25.7)	23.2 (21.6, 24.8)
Missings, N	0	5	5
Limonene [µg/m^3^]			
GM (95% CI)	12.1 (9.9, 14.8)	10.9 (8.9, 13.4)	11.5 (9.8, 13.5)
Missings, N	34	43	77

This table shows the descriptive analysis of the sample characteristics (GerES IV (2003–06), GerES V (2014–17), and pooled data). Percentages, arithmetic means, geometric means, 95% confidence intervals, standard deviations and medians were calculated by weighting the data and specifying the sampling points as cluster sampling probabilities. Absolute numbers were calculated unweighted.

AM, arithmetic mean; CI, confidence interval; GerES, German Environmental Survey; GM, geometric mean; N, absolute number of observations; SD, standard deviation.

The precautionary Guidance Values I (GV I) of the German Committee on Indoor Air were exceeded in two cases for toluene (GV I: 300 µg/m^3^)^33^, in five cases for xylene (GV I: 100 µg/m^3^)^34^, in one case for formaldehyde (GV I: 100 µg/m^3^)^35^, and in one case for limonene (GV I: 1000 µg/m^3^)^7^. To date, there is no GV I for benzene in the indoor environment, but a provisional guidance value of 4.5 µg/m^3^^36^, which was totally exceeded in 99 cases (see Supplementary Table S2 and Table S3 for socioeconomic and -demographic characteristics of the cases exceeding precautionary and provisional guidance values).

### Regression analyses

Table 2 summarizes the associations between the socioeconomic and -demographic variables and the indoor air pollutant concentrations. Our model demonstrated that an increase of one SD in equivalized disposable income was associated with a 5% decrease in benzene concentrations (exp(ß): 0.95; 95% CI 0.91, 0.99). No statistically significant association was observed between equivalized disposable income and the concentration of the remaining indoor air pollutants included in the analysis. All CIs for these effect estimates included the null effect (= 1.0). An increase of one SD in socioeconomic status was associated with a 10% decrease in benzene (exp(ß): 0.90; 95% CI 0.87, 0.94) and a 6% decrease in toluene concentrations (exp(ß): 0.94; 95% CI 0.89, 0.99). The CIs for the effect estimates of xylene (exp(ß): 0.97, 95% CI 0.92, 1.02), formaldehyde (exp(ß): 0.99, 95% CI 0.95, 1.03), and limonene (exp(ß): 0.96, 95% CI 0.90, 1.02) included the null effect.

**Table 2:** Associations between socioeconomic and -demographic variables and the selected volatile organic compounds.

Models	Fixed effects			τ_00_	τ_00_	τ_00_	ICC	Observations
	exp(ß)	95% CI	*p*	Point	Month	Year		
Benzene								
Income	0.95	(0.91, 0.99)	.015	.07	.23	.09	.554	1034
SES	0.90	(0.87, 0.94)	< .001	.07	.22	.09	.556	1037
History of migration	0.78	(0.68, 0.89)	< .001	.07	.23	.10	.563	1011
Toluene								
Income	1.00	(0.95, 1.06)	.995	.10	.13	.31	.462	1034
SES	0.94	(0.89, 0.99)	.019	.10	.13	.29	.451	1037
History of migration	0.87	(0.72, 1.06)	.170	.11	.13	.30	.459	1011
Xylene								
Income	1.03	(0.97, 1.09)	.325	.13	.12	.07	.342	1034
SES	0.97	(0.92, 1.02)	.258	.12	.12	.06	.333	1037
History of migration	0.95	(0.79, 1.15)	.603	.13	.12	.06	.343	1011
Formaldehyde								
Income	1.02	(0.98, 1.06)	.401	.08	.02	< .01	.342	1105
SES	0.99	(0.95, 1.03)	.739	.08	.02	< .01	.218	1109
History of migration	1.24	(1.07, 1.43)	.005	.09	.02	< .01	.231	1081
Limonene								
Income	0.97	(0.91, 1.04)	.395	.16	.89	.01	.542	1034
SES	0.96	(0.90, 1.02)	.194	.15	.88	.01	.538	1037
History of migration	2.02	(1.61, 2.55)	< .001	.14	.84	.01	.531	1011

This table shows the effect estimates, 95% confidence intervals and *p* values for the fixed effects, the variance explained by the random intercepts, the intraclass correlation coefficient and the absolute number of observations in the analysis. Income refers to equivalized disposable income. The reference group of the binary migration history variable is ‘having no parental migration history’. The random intercepts include the following number of cluster levels: 168 sampling points, seven data collection years and 12 data collection months.

SES, socioeconomic status; exp(ß), effect estimate; CI, confidence interval; ICC, Intraclass correlation coefficient; τ_00_, Variance of random intercepts.

If children and adolescents had a parental history of migration, our models showed a 24% increase in indoor air concentrations of formaldehyde (exp(ß): 1.24, 95% CI 1.07, 1.43) and a 102% increase of limonene concentrations (exp(ß): 2.02, 95% CI 1.61, 2.55). In contrast, benzene concentrations were 22% lower in households with a parental history of migration (exp(ß): 0.78, 95% CI 0.68, 0.89), compared to those without a parental history of migration. Please refer to Supplementary Table S4 for the results of our sensitivity analysis applying an alternative definition of migration background.

The Intraclass Correlation Coefficient (ICC) for the models ranged between 0.218 and 0.563. A considerable amount of variation in the outcome variable is due to the data structure, which is accounted for by the random intercept models.

### Subgroup analysis

**Fig. 1:**
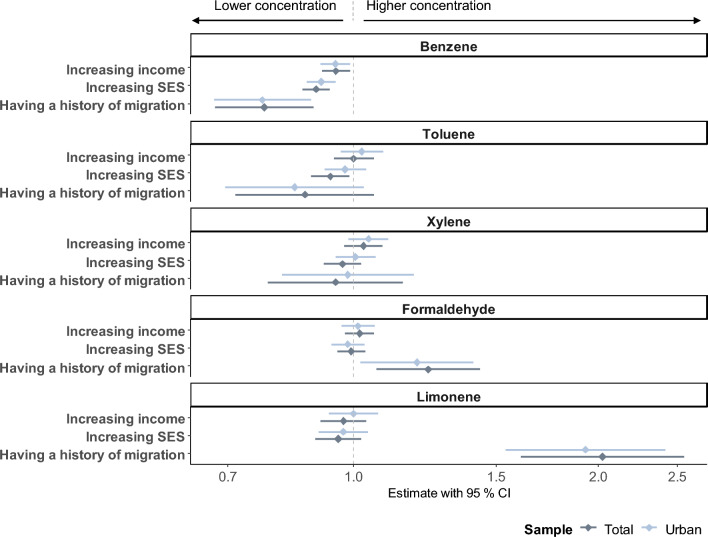
Effects between socioeconomic and -demographic variables with the selected volatile organic compounds in the total sample compared to the sample in urban areas. This figure plots the back-transformed regression estimates of the fixed effect (rhombus) with the 95% confidence intervals as calculated by the random intercept models on a logarithmic scale. Income refers to equivalized disposable income. SES, socioeconomic status; CI, confidence interval.

In our subgroup analysis we compared the estimates of the fixed effects between our total sample and the sample of households in municipalities with ≥ 20,000 inhabitants (Fig. 1). The effects were not exacerbated by municipality size. The magnitude of the aforementioned effects, pertaining to benzene and all three socioeconomic and -demographic variables, as well as for limonene and formaldehyde and a parental history of migration, were analogous to those observed in our previous findings. The effect of a one SD increase in socioeconomic status on toluene could not be verified for the sample in urban areas (exp(ß): 0.98; 95% CI 0.92, 1.04).

## Discussion

The primary objective of the present study was to quantify the association between equivalized disposable income, socioeconomic status and parental history of migration with the concentration of five selected indoor air pollutants in children and adolescents in Germany. Overall, five of the 15 models were consistent with our hypotheses, one showed an unexpected effect and nine were statistically insignificant.

As hypothesized, we found that on average a higher equivalized disposable income corresponded to a lower benzene concentration. In all other models where equivalized disposable income served as a fixed effect, we could not reject the null hypothesis. Our data support the alternative hypothesis that an elevated socioeconomic status relates to lower benzene and toluene concentrations. The other models using socioeconomic status as a fixed effect showed no statistically significant effects. Additionally, households of children and adolescents with a parental history of migration exhibited higher formaldehyde and limonene concentrations, as expected. Contrary to our assumption, having a parental history of migration was also associated with lower benzene concentrations.

When interpreting the models on associations of parental history of migration, the relatively small number of subjects classified as having a parental history of migration must be considered. Lower statistical power increases the uncertainty of the effect estimates and thus the possibility of false negative results. In our sensitivity analysis, we used the broader statistical concept migration background, which applies to twice as many cases as history of migration. We favour the somewhat stricter variant of parental history of migration because it depicts experiences of discrimination more accurately.

A secondary objective was to investigate, whether these associations may differ by the size of municipality. Our subgroup analysis revealed that the patterns observed in the more urban subsample, were highly similar to those in the total sample. However, the effect estimate of socioeconomic status on toluene was not statistically significant after we restricted the analysis sample to children and adolescents from more urban areas. Overall, socioeconomic and -demographic inequalities regarding indoor air pollution in the urban population do not appear to differ substantially from those observed in the population as a whole.

A number of studies have also investigated the distribution of VOC across socioeconomic and -demographic groups^23,24,26,28,37^. However, given the different indoor air pollutants and socioeconomic and -demographic variables, sufficient comparability only appears meaningful for certain studies. Subsequently, we provide an overview of the results of these studies that are of relevance for the contextualization of our findings.

In a French study, the chemical concentrations of formaldehyde and an index of benzene, toluene, ethylbenzene and xylene (BTEX) were measured during one week in 567 households between September 2003 and December 2005^23^. Bivariate comparisons of these indoor air pollutants between household income groups were not statistically significant. The authors ran a stepwise linear regression model treating formaldehyde (log-transformed) as the dependent variable and income as the independent variable, adjusting for various socioeconomic and -demographic variables, occupant activities, and building characteristics. Their results showed that on average, higher household income was associated with lower formaldehyde levels. In contrast, our analysis showed a null effect of equivalized disposable income and formaldehyde. However, we observed that children and adolescents with a parental history of migration are associated with living in homes with elevated formaldehyde concentrations, a factor not examined in the French study.

In another study, data collected between July and September 2008 examined 24-h concentrations of benzene, toluene and xylene in 50 children’s households in three South Korean cities^28^. The authors calculated general linear models to estimate the magnitude of the associations between these VOCs and average monthly expenses, adjusting for other socioeconomic and behavioral factors. None of these multivariable models showed a statistically significant association between monthly expenses and indoor air pollutants. If we assume monthly expenses as a proxy of equivalized disposable income, our results are mainly consistent with these findings. Our data also revealed no statistically significant associations between equivalized disposable income and toluene or xylene concentrations. In contrast to the previous study, we observed that higher equivalized disposable income corresponds to lower benzene concentrations.

In an earlier South Korean study from August 2001, benzene, toluene and o-xylene concentrations were quantified in 30 households in two cities^37^. The authors computed t-tests comparing VOC concentrations between participants residing in apartments versus those residing in low-income housing. Their results suggest that indoor concentration levels of benzene, toluene, and o-xylene were statistically significantly higher among participants residing in low-income housing, compared to those living in apartments. It is also possible to consider low-income housing as a proxy for a low equivalized disposable income or socioeconomic status. In this case, our results confirm the hypothesis that lower benzene concentrations are associated with higher equivalized disposable income and higher socioeconomic status. Furthermore, we also found that toluene is associated with higher socioeconomic status. In case of xylene, our results remained statistically non-significant.

Overall, the current state of research exhibits a lack of consistency with regard to the relationship between VOCs and socioeconomic and -demographic characteristics. These inconsistencies may be due to differences in (a) the target populations (e.g., space and time), (b) the recruitment (e.g., non-probability sample), (c) the measurement (e.g., measurement technique), (d) statistical model specifications (e.g., adjustment set), or (e) simply by chance (i.e., random error). However, such inconsistencies are not a general pattern among all indoor air pollutants. For instance studies have demonstrated that a low income is consistently associated with elevated levels of indoor air pollution with regard to PM^19–22^.

The present study is characterized by a number of strengths. To the best of our knowledge, this is one of the few studies to focus on inequalities in indoor air pollution rather than ambient air pollution and, the first in Germany. Moreover, the results have a high external validity, as our analysis is based on data from a random sample. Despite the age restrictions in our study sample (3–14 years), we are nonetheless confident that our results can be generalized to children and adolescents living in Germany. Compared to previous studies^20–24,26–29,37^, we were able to utilise a relatively large study sample by pooling several data sets. Furthermore, we employed validated measurement methods over a period of one week to reliably record indoor air pollutant concentrations. Finally, we considered spatial and temporal variations by utilising random intercept models in our data analysis.

When interpreting our results, some limitations need to be considered. Firstly, the associations we describe should not be interpreted causally. It is possible, that systematic errors such as selection bias, measurement/information bias, or confounding may be responsible for biasing the observed relationships^30^. A causal framework would be required to disentangle the complex nature of indoor air pollution inequalities^38,39^. These inequalities could be attributed to different mediation pathways. For example, low equivalized disposable income might cause higher benzene concentrations via occupants smoking behavior and/or via residing in close proximity to roads with high traffic density. But our descriptive analyses merely point to potential inequalities and are not sufficient to identify causal mediators. Causal inferences are beyond the scope of our descriptive study. Instead, we sought to quantify the strength and direction of the overall associations. The application of causal mediation analysis could, for example, facilitate the estimation of the natural indirect effect of low socioeconomic status on benzene concentrations through occupants smoking behavior^40^. These results could be used to target behavioral and/or structural prevention to protect the health of sensitive populations such as children and adolescents.

Secondly, it is possible that our results may be subject to bias due to factors related to measurement and information processing at design and analysis levels. For example, at the design level, data on socioeconomic and -demographic characteristics were self-reported by the parents. Such survey information may be recalled and/or reported inaccurately (under- or overreporting), potentially causing self-reporting bias. At the analysis level, there is a possibility of misclassification of the parental history of migration due to the definition we applied to group participants (yes = both parents born in another country than Germany, no = at least one parent born in Germany). This proxy does not perfectly reflect our underlying assumptions in the context of discriminatory experiences of parents, e.g. in the German housing market. The within-group heterogeneity of participants that were categorized as having a history of migration is large and thus there may have been considerable variation in their parents’ experiences of discrimination. Furthermore, reasons for discrimination might be manifold and the data used in our analyses does not allow an in-depth examination of differentiated processes of discrimination^41^. In addition, migration movements to Germany have increased in recent years and the social structure of people with a history of migration has changed since the data for our study was collected^42^. Therefore, the results can only be interpreted against the background of the social structure at the time of the data collection.

Another problem of potential misclassification is the dichotomization of the municipality size variable for the subgroup analysis (municipalities with < 20,000 inhabitants vs. municipalities with ≥ 20,000 inhabitants). This procedure at the analysis level could be criticized as masking hidden heterogeneity within the rural and urban groups. More fine-grained measures for the rural–urban continuum may be more appropriate in future research^43^. However, given the exploratory nature of the subgroup analysis that we calculated for our secondary research question, a dichotomization seems sufficient.

Thirdly, selection bias is another type of systematic bias which may influence the validity of our findings. Although the selection of sampling points and the recruitment of test subjects is carried out using a multi-stage randomized procedure, the ultimate decision regarding participation in the KiGGS study and subsequent participation in the GerES study is made by the families themselves. If they systematically differentiate between those who participate in the studies and those who do not, a self-selection bias may occur. To counter this issue, we applied sampling weighting procedures to match our sample to the German census taking participation probabilities of different socioeconomic and -demographic groups into account.

In light of the strengths and limitations of our study, we can derive the following implications from our findings. It is encouraging to note, that despite some exceedances the geometric mean concentration levels we observed for the total study sample were well below the precautionary GVs I and the provisional guidance value by the German Committee on Indoor Air. Fortunately, we also found that the geometric mean of the five selected substances examined here were lower in GerES V (2014–2017) than in GerES IV (2003–2005). However, our results also show that inequalities in indoor air pollution are apparent in the present sample. For example, children and adolescents with a parental history of migration tend to live in homes where limonene pollution is more pronounced. Additionally, indoor air benzene concentrations are elevated among children from lower socioeconomic status. The latter finding is of particular public health relevance, as benzene is a genotoxic carcinogen with no safe level of exposure^6^. Future research could go beyond investigating benzene variability in different social strata (= exposure variation) and quantify possible differences in the effect of a given benzene exposure on health outcomes in different socioeconomic groups (= effect modification)^44^.

Although not all of our modelling showed a consistent picture across the five selected VOCs, it can be reasonably concluded that children and adolescents in Germany are affected by inequalities in indoor air pollution. Even small effect sizes can have a large impact when applied to the entire target population. The most recent census, conducted in 2022, indicated that there were around 14.6 million persons under the age of 18 living in Germany^45^. The majority of time is spent indoors for these children and adolescents, rendering them highly susceptible to indoor air pollutants and offering minimal opportunity to alter their living environment.

Elevated concentrations of VOCs may co-occur. Even if the guidance values for single pollutants are rarely exceeded, combined human exposures to mixtures of different VOCs may have additive or synergistic health effects^46^. Future analyses could investigate the potential cumulative concentrations of the more than 70 different VOCs measured in GerES. Moreover, it is evident that the inequalities we observed in the five selected indoor air pollutants are not isolated, but rather add to the broader inequalities present in outdoor environments (e.g. ambient air pollution)^15,16^. Children from families with low household income, low socioeconomic status or a history of migration are also exposed to a variety of other structural disadvantages in Germany. These include unequal opportunities in education^47^, on the housing market^48^, on the labor market^49^, and in terms of health^50^. For policymakers, this signifies that social inequalities persist from childhood to adulthood and that a holistic policy is needed to counteract these complex problems.

It is imperative that environmental justice is incorporated into all policies. For example, the Federal Ministry responsible for urban development and construction could consider including environmental justice in the catalogue of the German Building Code as one of the interests to be taken into account in urban land use planning^51^. In the context of construction and housing, the responsible planners, authorities and property developers should consider the potential for indoor and outdoor sources of VOCs at an early stage and reduce them as much as feasible. In addition, construction products should not be allowed to emit carcinogenic substances as a precautionary measure. With regards to the European market, the European Commission should set appropriate minimum standards for all indoor construction products.

Disadvantaged families have less means to change their housing situation in the case of disproportionate pollution. Healthy living environments, even beyond housing (e.g., kindergartens, schools, or workplaces), should be an established standard. Our subgroup analysis also make it clear that the associations found are consistent in the overall sample and in the urban sub-sample. Urban planning measures for improved environmental justice should therefore reach not only German cities, but all regions. Urban and spatial planners should consider not only the physical environment but also the socio-spatial structures of the neighborhood, including pollutant emitters such as petrol stations or nail salons.

## Conclusion

Environmental justice advocates can find some confirmation of indoor air pollution inequalities in Germany from our findings. Children and adolescents who are already socially disadvantaged may also be disproportionately exposed to VOCs. However, we also emphasize that the patterns of inequality are not as straightforward or consistent across all selected indoor air pollutants and socioeconomic and -demographic characteristics. Future explanatory research could substantiate our descriptive findings by applying causal methods and quantifying direct, indirect and total causal effects, thereby making an important contribution to protecting public health against indoor air pollution.

## Methods

### Data sources

The German Health Interview and Examination Survey for Children and Adolescents (KiGGS) provided the socioeconomic and -demographic information for this study. The Robert Koch Institute (RKI) designed the KiGGS study as a representative, nationwide survey with the target population of 0 to 17-year-olds in Germany. At baseline, a total of 17,641 participants from 167 municipalities (sampling points) were enrolled between May 2003 and May 2006^52^. In KiGGS Wave 2, the RKI collected new cross-sectional data from 15,023 children and adolescents from the original 167 KiGGS sampling points between September 2014 and June 2017^53^.

The German Environmental Survey for Children 2003–06 (GerES IV) and the German Environmental Survey for Children and Adolescents 2014–2017 (GerES V) were random subsamples of KiGGS Baseline and KiGGS Wave 2 respectively^54,55^. The German Environment Agency (UBA) designed GerES to collect representative data on the population’s exposure to health-relevant environmental influences and pollutants. In GerES IV, data of 1790 children and adolescents, aged between 3 and 14, was collected in 150 of the sampling points^56^. In GerES V, data of 2,294 children and adolescents, aged between 3 and 17, was collected in all 167 sampling points^57^. For our analysis, we used a sub-sample of 3 to 14-year-old children and adolescents from GerES V for the purpose of comparability with GerES IV. The sampling points of both studies were matched via a key variable. In one case, it was not possible to assign the sampling point ID between GerES IV and GerES V without any doubt. Our study sample therefore comprised 168 sampling points.

The Ethics Committee of the Charité University Hospital of the Humboldt University of Berlin approved of GerES IV and KiGGS Baseline (No. 101/2000). The Hanover Medical School’s ethics committee approved KiGGS Wave 2 (No. 2275-2014) and the ethics commission of the Berlin Chamber of Physicians approved GerES V (Eth-14/14). All studies were approved by the German Federal Commissioner for Data Protection and Freedom of Information.

### Socioeconomic and -demographic variables

For this analysis, we used parent-reported data on household income, socioeconomic status and history of migration from the KiGGS Baseline survey and KiGGS Wave 2. The household income was operationalized by the needs-adjusted net household income considering the household composition and age of the household members (equivalized disposable income, metric)^58^ based on the modified equivalence scale of the Organisation for Economic Co-operation and Development^59^. Accordingly, the equivalized disposable income was calculated as follows:

$$Equivalized\,Disposable\,Income = \frac{Net\,Household\,Income}{{Needs{\text{-}}Adjusted\,Household\,Size}}$$

where the net household income was collected as the total income of all household members (including parental allowance, child benefits, etc.) after deducting taxes and social contributions and where:

$$\begin{aligned} & Needs{\text{-}}Adjusted\,Household\,Size \\ & \quad = 1 + \left( {0.5 \times Number\,of\,Additional\,Adults\,and\,Children\,14\,years\,or\,older} \right) \\ & \quad + \left( {0.3 \times Number\,of\,Children\,under\,14\,years} \right) \\ \end{aligned}$$

This ensured that incomes are comparable across households of varying sizes, as larger households experience cost-saving benefits (economies of scale), such as shared housing and household appliances. Additionally, children are assumed to have lower financial needs compared to adults.

Socioeconomic status was operationalized as a summative index score (range 3–21), which had been calculated from the parents’ education, occupation and equivalized disposable income. Parents’ education was measured using the CASMIN classification (Comparative Analyses of Social Mobility in Industrial Nations)^60^. Occupational status was measured using the ISEI classification (International Socio-Economic-Index of Occupational Status)^61^. All three dimensions were assigned point scores, which were then summed to create the socioeconomic status score. A more detailed description of the calculation and point score assignment can be found elsewhere^58^. The methods used to assess equivalized disposable income and socioeconomic information were comparable between KiGGS Baseline and KiGGS Wave 2.

In Germany, the statistical category migration background is typically used to summarize various forms of migration, origins and differences in spoken languages^62^. A child or adolescent has a migration background if the person or at least one of his or her parents did not acquire the German citizenship at birth^63^. Despite its popularity, the concept of migration background has been criticised for numerous shortcomings^62,64,65^ and it is therefore recommended to operationalise migration in the context of the respective research question and explicitly think about the assumed underlying mechanisms^66^. As we intended to apply a marker that more precisely reflects potential experiences of discrimination, we decided to calculate the variable ‘history of migration’. A history of migration was assigned to participants, if both parents were born in another country than Germany (= yes). If at least one parent was born in Germany, observations were defined as having no history of migration (= no). Our definition of history of migration is therefore narrower than migration background. For single parents, we used the details of the person with whom the child lives. This data was not assessed uniformly across KiGGS Baseline and KiGGS Wave 2. For GerES IV (KiGGS Baseline), we used the information on country of birth of the mother and/or father. For GerES V (KiGGS Wave 2), we used information on the immigration group of the mother and/or father (which contains the information whether or not a parent was born in Germany).

### Indoor air pollution variables

The five VOCs we selected to represent indoor air pollution are benzene, toluene, xylene, formaldehyde, and limonene. They are of public health relevance and represent a variety of indoor air pollution sources. They were measured in GerES IV and V with the same techniques. Indoor air concentrations of VOCs were quantified through the utilization of passive sampling techniques. This approach offers significant advantages in a domestic environment, particularly due to the low sampling effort and minimal inconvenience to participants. Sampling was conducted in the room where, according to parent reported information, the child or adolescent typically spent the majority of their time indoors. In 95% of cases, the child’s bedroom was identified as the primary location. The concentrations of the measured VOCs and aldehydes reflect the emissions under actual conditions of use in the rooms. The passive sampling was conducted for a period of seven days under conditions of real-use, whereby the occupants ventilated their dwelling in accordance with their usual practices. The home visits for the cross-sectional measurements took place in all 12 months of the year. For the calculation of statistical parameters, data points below the limit of quantification (LOQ) were assigned a value equal to two thirds of the LOQ. Xylene was the calculated sum of m-, p- and o-xylene. For a detailed explanation of methodologies regarding sample preparation and analysis, the reader is referred to the methodology and materials as described elsewhere^11,67^.

### Trend weights

Trend weights were added to reflect the GerES IV data to the sociodemographic characteristics of the general population of children and adolescents living in Germany between 2014 and 2017. The trend weights included the study population of children and adolescents between the ages of 3 and 14. They were previously calculated (see Birmili et al. (2021) for more information)^67^.

### Data curation

After the variables had been compiled from the original data from GerES IV and GerES V and the respective corresponding datasets from KiGGS, we obtained a pooled database with 4084 participants. The study sample was further specified applying eligibility criteria. First, only observations were included in which at least one of the social variables and at least one of the indoor air variables was specified (N = 1221). Second, observations without information on the trend weight were excluded (N = 104). These observations consist of participants of GerES V who were between 15 and 17 years old.

### Statistical analysis

We used descriptive statistics to summarize the characteristics of the GerES IV and GerES V samples as well as the pooled data. For categorical variables, we reported the absolute numbers (N) and relative proportions (%) of observations. We calculated arithmetic means with SD, medians and geometric means with 95% CI for metric variables. In order to describe the outcomes of the present study not only on an average level, we also investigated how many cases exceed the guidance values of the selected VOCs.

For each of the five indoor air pollutants, linear multilevel regression models with random intercepts were calculated to quantify the associations with the fixed effects (a) equivalized disposable income, (b) socioeconomic status or (c) history of migration. To enable comparability of the coefficients, equivalized disposable income and socioeconomic status were scaled and centred (z-transformation). Non-nested random intercepts were included for (a) the sampling point to incorporate an approximation of the spatial variance, (b) the sampling year to cover temporal trends, such as legislative regulations or shifts in income distribution, and (c) the sampling month to reflect seasonal changes in indoor and outdoor air pollutant sources. The response variables were log-transformed to reduce heteroscedasticity and skewness of the distribution of residuals. Effect estimates and 95% CI were reported on the original scale using exponentiation. Therefore, the reported estimates (exp(β)) can be interpreted as proportionate change in the expected geometric mean of the original variable. For the z-transformed variables equivalized disposable income and socioeconomic status, an increase by one SD was associated with a proportionate change in the outcome variable. For the variable ‘history of migration’, the proportionate change was associated with ‘having a parental history of migration’, compared to ‘having no parental migration history’. The significance level (α) for the statistical tests was set at 0.05.

We conduct all analyses applying trend weights^67^. Absolute numbers were calculated unweighted^68^.

For the subgroup analysis, we calculated random intercept models, as described above, for the study sample of children and adolescents living in municipalities with ≥ 20,000 inhabitants (N = 838). We chose this cut-off for the sake of consistency and comparability with previous research^69^. For the sensitivity analysis, we used the migration background instead of history of migration in the random intercept models for migration. In this way, we wanted to analyse the robustness of the effect estimates in relation to the definition of migration.

All statistics were calculated using R version 4.4.1 and RStudio version 2024.4.2.764. The R packages we applied include: broom, tidyverse, hablar, haven, Hmisc, wCorr, lmerTest, jtools, survey, weights and performance.

## Supplementary Information

Supplementary Information.

## Data Availability

Researchers interested in accessing the KiGGS Baseline, KiGGS Wave 2, or GerES V datasets can request them from the ‘Health Monitoring’ Research Data Center at the Robert Koch Institute (RKI) (https://www.rki.de/DE/Content/Forsch/FDZ/FDZ_node.html). The GerES IV dataset is available upon request from the German Environment Agency (https://www.umweltbundesamt.de/themen/gesundheit/belastung-des-menschen-ermitteln/umwelt-survey/umwelt-surveys-1985-bis-2006).
