# Supplementary material for: Indoor air pollution inequalities among children and adolescents in Germany: an analysis of repeated cross-sectional data from GerES and KiGGS
Source: Sci Rep. 2025 Jun 20;15:20119. doi: 10.1038/s41598-025-04278-9 (PMC12181257; doi:10.1038/s41598-025-04278-9)
Supplement: Supplementary file 1 — Supplementary Information. [file 41598_2025_4278_MOESM1_ESM.pdf]

Supplementary Material

Indoor air pollution inequalities among children and adolescents in Germany: An analysis of repeated cross-sectional data from GerES and KiGGS

Jascha Wiehn, Sarah Tietjen, Florian Beese, Wolfram Birmili, Christiane Bunge, Anja Daniels, Annika Fernandez Lahore, Domenica Hahn, Marike Kolossa-Gehring, Ronny Kuhnert, Aline Murawski, Julia Waldhauer, Dario Zocholl, & André Conrad

Supplementary Figure S1. Income distribution of the study sample.

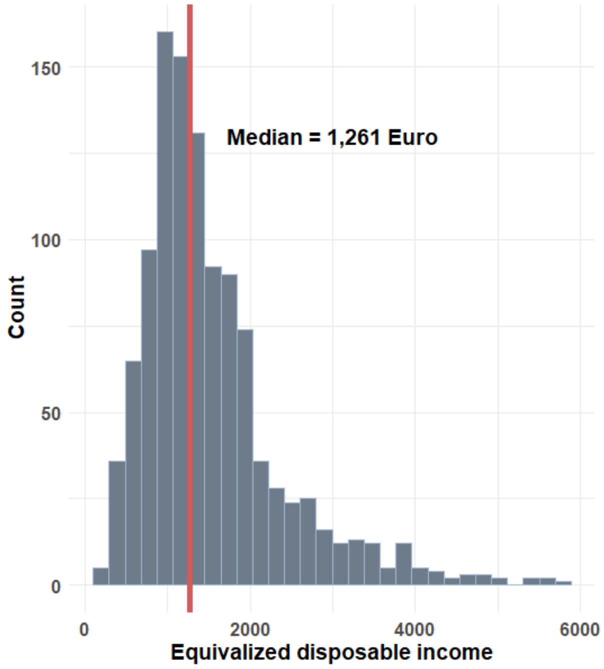

Note. This histogram shows the distribution of the equivalized disposable income in the study sample. The red line indicates the median income of 1,261 Euro.

Supplementary Table S2. Socioeconomic and -demographic features of the nine cases with precautionary Guidance Values I exceeding for Toluene, Xylene, Formaldehyde and Limonene.

	**Municipality size**	**Equivalized disposable income**	**Socioeconomic status**	**Having a history of migration**
Toluene				
> 300 µg/m³	≥ 20,000 inhabitants	1408	16	No
	≥ 20,000 inhabitants	940	13	No
Xylene				
> 100 µg/m³	< 20,000 inhabitants	1457	9	No
	< 20,000 inhabitants	1130	11	No
	≥ 20,000 inhabitants	2174	20	No
	≥ 20,000 inhabitants	1526	15	No
	≥ 20,000 inhabitants	1667	17	No
Formaldehyde				
> 100 µg/m³	≥ 20,000 inhabitants	2083	15	No
Limonene				
> 1,000 µg/m³	≥ 20,000 inhabitants	1085	10	Yes

Note. This table shows the socioeconomic and -demographic features of households in which the precautionary Guidance Values I for Toluene, Xylene, Formaldehyde and Limonene of the German Committee on Indoor Air were exceeded.

Supplementary Table S3. Descriptive characteristics of the households with provisional guide value exceeding for benzene.

	**Benzene > 4.5 [µg/m³] (N = 99)**
Municipality size, N (%)	
< 20,000 inhabitants	32 (39.4)
≥ 20,000 inhabitants	67 (60.6)
Missings, N	0
Equivalized disposable income [Euro]	
AM (SD)	1,122.1 (605)
Median	966
Missings, N	1
Socioeconomic status	
AM (SD)	10 (4)
Missings, N	0
History of migration, N (%)	
No	96 (99.3)
Yes	2 (0.7)
Missings, N	1

Note. This table summarizes the descriptive characteristics of the 99 cases in which the provisional guide value for benzene was exceeded. Percentages, arithmetic means, standard deviations and median were calculated by weighting the data and specifying the sampling points as cluster sampling probabilities. Absolute numbers were calculated unweighted.

Abbreviations. AM, arithmetic mean; N, absolute number of observations; SD, standard deviation.

Supplementary Table S4. Sensitivity analysis on the associations between migration background and the selected volatile organic compounds.

Models	Fixed effects exp(ß)	95 % CI	p	τ_{00} Point	τ_{00} Month	τ_{00} Year	ICC	Observations
Benzene								
Migration background	0.97	(0.88, 1.07)	.569	.07	.23	.10	.564	1,035
Toluene								
Migration background	0.97	(0.85, 1.11)	.663	.10	.13	.30	.460	1,035
Xylene								
Migration background	1.00	(0.87, 1.14)	.947	.13	.12	.06	.339	1,035
Formaldehyde								
Migration background	1.05	(0.95, 1.16)	.339	.08	.02	<.01	.229	1,106
Limonene								
Migration background	1.49	(1.27, 1.75)	<.001	.14	.85	.01	.530	1,035

Note. This table shows the effect estimates, 95 % confidence intervals and p-values for the fixed effects, the variance explained by the random intercepts, the intraclass correlation coefficient and the absolute number of observations in the analysis. The reference group of the binary migration background variable is 'having no parental migration background'. Migration background was defined as the individual or at least one of his or her parents did not acquire the German citizenship at birth. The random intercepts include the following number of cluster levels: 168 sampling points, seven data collection years and 12 data collection months.

Abbreviations. exp(ß), effect estimate; CI, confidence interval; ICC, Intraclass correlation coefficient; τ_{00}, Variance of random intercepts.
